# Pregnancy and Delivery Management With Recombinant Factor VIIa in a Glanzmann Thrombasthenia Patient: A Case Report

**DOI:** 10.7759/cureus.22570

**Published:** 2022-02-24

**Authors:** Leonardo Enciso, Rafael L Aragón-Mendoza, Laura A León, Carrie G Torres-Torres

**Affiliations:** 1 Hematology, Hospital Universitario Nacional de Colombia, Bogotá, COL; 2 Hematology, Hospital Universitario de La Samaritana, Bogotá, COL; 3 Maternal-fetal medicine, Hospital Universitario de La Samaritana, Bogotá , COL; 4 Internal Medicine, Fundación Universitaria Juan N. Corpas, Bogotá, COL

**Keywords:** platelet transfusion, platelet refractoriness, recombinant activated factor vii, pregnancy, glanzmann thrombasthenia

## Abstract

The management of pregnancy and delivery in patients with Glanzmann thrombasthenia requires platelet transfusion and recombinant activated factor VII. We report two successful pregnancies in a single patient and propose a protocol for monitoring and treatment. The urgent need for controlled trials and other epidemiological studies is also underscored.

## Introduction

Glanzmann thrombasthenia (GT) is a rare inherited disorder of platelet aggregation, with an estimated incidence of one in 1,000,000, and autosomal recessive inheritance, affecting males and females equally [1]. The mechanism of abnormal platelet aggregation is the absent or reduced expression of platelet glycoprotein IIb/IIa or the expression of a dysfunctional glycoprotein. Normal platelets express near 50,000 copies of this glycoprotein at their membrane. Conformational glycoprotein changes lead to fibrinogen binding that triggers cytoskeletal changes, granule secretion, and fibrin clot stabilization [2]. The treatment of this disease requires antifibrinolytic agents and platelet transfusions, with the risk of developing antibodies against the missing glycoprotein and platelet transfusion refractoriness [3]. The use of recombinant activated factor VII (rFVIIa) seems to be an alternative to these patients and even in those without previous platelet transfusions or refractoriness, with efficacy rates greater than 80% [4]. The management of pregnancy and delivery in GT-affected female patients includes platelet transfusion, iron replacement therapy, and rFVIIa, but most information comes from individual case reports and case series [5]. Here we report our experience with two pregnancies in a single female patient and the use of rFVIIa to control bleeding.

## Case presentation

A 21-year-old female patient presented to the outpatient hematology clinic with an epistaxis and gum bleeding history beginning in infancy. She also reported menorrhagia from menarche and received multiple packed red blood cells transfusions because of symptomatic anemia. The patient lives in a rural zone with limited health services and only has sporadic internal medicine consultations. At the first visit, she was in week 6 of her first pregnancy and persisted with episodic epistaxis and gingival bleeding. The review of the coagulation test revealed that partial thromboplastin time and prothrombin time were within normal limits, and the platelet count was in the normal range. Platelet light transmission aggregometry found absent aggregation with all physiologic agonists and a normal agglutination response to ristocetin. A GT was then confirmed, and the patient continued follow-up in the ambulatory service. Flow cytometry to evaluate platelet glycoprotein expression was not available. The patient began treatment with oral iron supplementation and tranexamic acid mouth rinses with partial control of symptoms. No platelet transfusion was needed.

At week 20, a complete blood count revealed a drop in hemoglobin levels to 6.7 g/dL. Vital signs were stable, but the patient required admission to the obstetric unit given the low compliance with the ambulatory treatment. At admission, her blood pressure was 129/75 mmHg, heart rate was 70 beats per minute, respiratory rate was 20 breaths per minute, and temperature was 37°C. According to gestational time, uterine height was 20 cm on physical examination. Fetal heart rate was 127 beats per minute, and fetal well-being was confirmed. Parenteral iron therapy and two packed red blood cells were given before discharge. A treatment protocol that specifies the timing of platelet transfusion and the schedule of rFVIIa was specified and is present in Table 1.

**Table 1:** Protocol for monitoring and treatment NSAIDs, non-steroidal anti-inflammatory drugs

Variable	Recommendation
Prenatal controls, biophysical profile, and monitoring of fetal well-being	Week 13-28 every 15 days
	Week 36-40 weekly
Vaccination	Hepatitis B
Platelet transfusion	
Component	Isogroup platelets
Indications	Antenatal, surgical intervention, hemorrhage, hemodynamic instability, threat of fetal-placental unit
	Perinatal during delivery, when the cervix was 8-9 cm dilated
	Postnatal 12 hours after postpartum
Doses	1 apheresis unit, 4-6 individual units, 1 platelet pool
Recombinant activated factor VII	
Indications	Surgical intervention, cesarean section, hemorrhage, hemodynamic instability
Doses	30-90 mg/kg. The dose can be repeated after two hours in no bleeding control.
Analgesia	No NSAIDs
Delivery route	Preferred: vaginal, 40 weeks
	Alternative: Cesarean section, 37 weeks
Thromboprophylaxis	Not indicated
Contraceptive method	Hormonal intrauterine device.

At week 39.4, the patient initiated active phase of labor. After transfusion of six isogroup platelet units, the patient delivered a healthy female neonate. The patient had profuse vaginal bleeding in the immediate puerperium, and administration of 2 mg of rFVIIa led to complete hemorrhage control. She was discharged one week after delivery with a hemoglobin level of 10.5 g/dL and a platelet count in the normal range.

Five years later, the patient returned to our hospital at week 33.4 of her second pregnancy. The patient has poor prenatal control and did not report ambulatory hematology consultations. She reported multiple admissions between conceptions because of abnormal uterine bleeding and hematuria requiring multiple red blood cell transfusions. Her blood pressure was 128/84 mmHg, heart rate was 84 beats per minute, respiratory rate was 17 breaths per minute, temperature was 36.2°C, and saturation was 94%, with a grade II / IV systolic murmur in pulmonary focus. Uterine height was 33 cm, with a single live fetus in longitudinal breech presentation and a fetal cardiac frequency of 134 beats per minute without uterine activity. In week 37, a cesarean section was performed after transfusion of six isogroup platelet units and 2 mg of rFVIIA. Tubal sterilization was also performed. In the immediate puerperium, during surveillance in the intensive care unit, she received platelet transfusion every 12 hours for the first 36 hours and 2 mg of rFVIIa 12 hours after surgery to achieve bleeding control, along with transfusion of five packed red blood cells units. The patient developed severe preeclampsia and required oral and intravenous antihypertensive therapy. After achieving control of blood pressure, the patient was discharged with a hemoglobin level of 10.7 g/dL and a platelet count in normal limits.

## Discussion

Inherited platelet disorders are rare diseases. Hemorrhagic manifestations can be severe and life-threatening, and the diagnosis requires specialized methods and a high index of clinical suspicion. Broadly, platelet disorders comprise disorders of the platelet number (e.g., MYH9 syndrome and TAR syndrome), severe disorders of platelet function related to a deficiency on platelet glycoproteins (e.g., GT and Bernard-Soulier syndrome), disorders of receptors and signal transduction (e.g., Thromboxane synthase deficiency), disorders of platelet granules (e.g., idiopathic dense-granule disorder), and disorders of phospholipid expression (Scott syndrome) [6]. The bleeding phenotype of patients with GT includes epistaxis, gum bleeding, menorrhagia, gastrointestinal and mucosal bleeding, and, in rare cases, intracranial hemorrhage [1]. The diagnosis is based on platelet light transmission aggregometry with aggregation only with ristocetin and absent aggregation with other agonists. Our patient had a classical clinical picture with preponderant mucocutaneous bleeding and menorrhagia, and platelet aggregometry confirmed the diagnosis.

The use of flow cytometry to detect glycoprotein-deficient expression is helpful but does not detect type III, a functional disorder with normal levels of expression of the IIb/IIIa glycoprotein. The iron deficiency is related to abnormal bleeding and is not corrected, leading to the need for packed red blood cells, as was seen in our patient. The management of pregnancy and delivery in GT patients is complex and requires a multidisciplinary approach. In the absence of clinical trials, the evidence supporting most interventions proceeds from case series and individual case reports. Our proposed protocol includes the time and frequency of antenatal visits, the indication and dose of platelet transfusions, the indication and dose of rFVIIIa, the analgesia and antithrombotic prophylaxis, the delivery route, and the discussion of contraception method. Even in the absence of clinical trials, this protocol is based on the information of management registered in case series and a systematic review [5,7]. In our context, the determination of platelet alloimmunization and the transfusion of HLA-matched platelets are not possible. The support for using rFVIIa in this patient and in patients without platelet antibodies came from registry data and a surveillance database that found an efficacy rate of 79% and 88% in the treatment of bleeding episodes and the prevention of surgical bleeding, respectively [4]. The recommended dose is 90 mcg/kg, which can be repeated at a two-hour interval. A series of 65 pregnancies in 34 women found severe bleeding leading to red blood cell transfusions in 50% of the 17 patients affected with GT despite the administration of platelet transfusions and antifibrinolytic therapy, suggesting that the use of rFVIIa could be an option.

## Conclusions

In conclusion, we reported two successful pregnancies and delivery in a single patient and reported our treatment protocol using platelet transfusions and rFVIIa. The absence of clinical trials limits a systematic approach, leading to clinical decision-making based on a low evidence level.
